# Through the lens of patients: a photovoice study exploring inpatients’ sense of safety and well-being in a new Norwegian mental health facility

**DOI:** 10.1186/s40359-025-03375-8

**Published:** 2025-09-10

**Authors:** Anne Hagerup, Carina Ribe Fernee, Helle Wijk, Göran Lindahl, Sepideh Olausson

**Affiliations:** 1 Institute of Health and Care Sciences, Sahlgrenska Academy, University of Gothenburg, Box 457, Gothenburg, 405 30 Sweden; 2 Inland School of Business and Social Sciences, University of Inland Norway, Lillehammer, Norway; 3 Department of Child and Adolescent Mental Health, Sørlandet Hospital, Kristiansand, Norway; 4 Department of Sport Science and Physical Education, University of Agder, Kristiansand, Norway; 5 Department of Quality Strategies, Region Västra Götaland, Sahlgrenska University Hospital, Gothenburg, Sweden; 6 Department of Architecture and Civil Engineering, Chalmers University of Technology, Gothenburg, Sweden; 7 Division of Construction Management, Department of Architecture and Civil Engineering, Chalmers University of Technology, Gothenburg, Sweden; 8 Department of Anesthesiology and Intensive Care/Sahlgrenska, Sahlgrenska University Hospital, Gothenburg, Sweden

**Keywords:** Biophilic design, Mental health facilities, Psychiatric patients, Phenomenological hermeneutics, Photovoice, Safety, Supportive design, Well-being

## Abstract

Patients’ sense of safety and well-being may be affected in numerous ways while being cared for in hospitals. Often, feelings of alienation arise, as private spaces like the home are inaccessible. One aspect that impacts patients’ safety and well-being is the design of the physical care environment. In this study, we sought to understand the impact of the physical environment and interpret the meanings of the care environment and its contributions to inpatients’ sense of safety and well-being. Fourteen adult patients were being cared for in a new mental health facility where a key feature is the focus on supportive and biophilic design and access to nature. Data were generated using a photovoice methodology (i.e., photos were taken by the participants themselves, followed by individual interviews). We adopted a phenomenological hermeneutic approach to research lived experiences and analyzed the interviews. Three major themes emerged: *being sheltered*, *feeling cared for and dignified*, and *being held by nature*. We found that the patients were able to find calmness, to land, and to rest in the new mental health facility because of the supportive design. Therefore, they were more open to therapeutic interventions that might make the recovery process faster. The insights from this study underscore the importance of prioritizing design features that address the various and holistic needs of patients in mental health care.

## Introduction

In Southern Norway, a new mental health facility inspired by nature and biophilic design—Sørlandet Hospital Mental Health Clinic [Nybygg Psykisk Helse Kristiansand], hereafter NPK for short—was completed in 2023. The concept of a supportive environment combined with biophilic design is believed to be foundational in addressing future health care needs and thus guided the design and construction of NPK. Biophilic design is “the deliberate attempt to translate an understanding of the inherent human affinity to affiliate with natural systems and processes – known as biophilia – into the design of the built environment.” ([34], p. 3). NPK has an attractive appearance and embodies the idea of nature’s calming effect on people. It aims to ensure that patients receive the best and most effective treatment available. A key feature is the strong focus on the therapeutic use of nature that surrounds the building and the nearby outdoor areas. In this study, as part of a larger project, we examine inpatients’ experiences of being cared for in this new facility and determine whether the environment supports their sense of safety and well-being.

Humanistic and holistic care can cultivate a broader understanding of health. As such, it is crucial to revisit Gadamer’s [17] idea that health is not merely the absence of illness but a state of wholeness and harmony in life. Additionally, it is necessary to explore how the built environment can support health within this broader perspective. This argument is particularly relevant for patients in mental health care. Individuals with mental health challenges form a diverse and vulnerable group, often facing stigma, shame, and anxiety when admitted to inpatient care [2]. Given the vulnerability of this patient group, the design of mental health hospitals should be tailored to address the specific needs of these individuals by creating spaces that promote well-being and recovery while fostering a sense of safety and dignity [1].

## Background

Patients admitted to mental health facilities often experience stress and anxiety [67]. A patient’s lifeworld is often impacted by the loss of personal agency and the inability to maintain a sense of self. The individual choices and private space that a home may represent are no longer available, and fear in conjunction with unpleasant procedures may negatively shape patients’ experiences and even worsen the situation [12]. In this context, researchers argued that if patients experience a hospital building as safe and as places where affordance is realized, they also feel an increased sense of well-being and positive health benefits [28, 31, 44], as well as better treatment outcomes [67]. According to Marberry et al. [43], purposefully built health care facilities have become a key component in today’s design of buildings, and an awareness has risen regarding the impact of care facilities on people’s health and well-being.

The environment can play an important role in patients’ recovery, boosting staff morale and enhancing the quality of life of both user groups [10, 31, 49]. For example, patients have highlighted lockable single rooms, physical activities, activities that allow patients to be someone else rather than “just” a patient for a moment, access to the outdoors, no rattling keys or echoing corridors, and permanent staff in a facility to increase their sense of safety and well-being [25, 51, 59]. Additionally, hospital environments with access to gardens is reported to reduce anxiety for patients and the level of stress for both patients and staff [1, 8, 9, 25, 33, 55, 59].

The concept of “well-being” and health in connection to built environments, such as how environmental qualities may impact well-being, has received increasing attention over the years [3, 22]. Well-being can be defined in numerous ways, depending on the discipline [27]. Within psychology, well-being can be defined as *“a state of happiness and contentment, with low levels of distress, overall satisfactory physical and mental health, or a good quality of life”* [4]. However, Galvin and Todres [18] argues that something is missing from contemporary health care: a need to humanize health and social care by linking well-being to caring. This underscores the importance of exploring how physical environments support individuals struggling with mental health challenges from a subjective perspective, particularly by emphasizing inpatiens’ lived experiences of being cared for in mental health facilities designed with the best intentions to be supportive. Accordingly, the aim of this study is to understand the impact of the physical environment and interpret the meanings of the care environment and its contributions to inpatients’ sense of safety and well-being.

The following research questions guided this study:

1. What are patients’ experiences of their care environment?
2. How does the built environment, both the indoors and the outdoors, impact the sense of safety and well-being of patients voluntarily admitted to inpatient mental health care?
3. Does nature play a role in the environment of the mental health facility? If so, in which ways does nature seem to impact patients’ experiences?

## Theoretical standpoints

Building on clinical and environmental psychology, this study focuses on patients’ experiences and their sense of safety and well-being when voluntarily admitted to a new mental health facility. The theoretical standpoints are grounded in the research on supportive environments [60]. In the context of this study, a supportive environment refers to *“environmental characteristics that support or facilitate coping and restoration with respect to the stress that accompanies illness and hospitalization”* ([62], p. 53). Indeed, being admitted to a mental health facility may be a stressful event for many patients, which in turn can negatively impact on both psychological and behavioral aspects of their care. Supportive design improves well-being and stress reduction and hence should be more intentionally used in mental health care. Biophilic design is associated with decreased levels of stress.

[63] and promotes calmness and relaxation [35, 36]. In nature, mental noise can be reduced and attention improved [32], creating a state of being where self-reflection and contemplation is more accessible.

### Study design

This study employed a qualitative methodology, specifically a phenomenological hermeneutic approach [42] and photovoice [66]. The field of phenomenological hermeneutics seeks to reveal the lived experiences of phenomena in a lifeworld. The phenomenon studied was experiences of the built environment and its contribution to inpatients’ sense of safety and well-being. The phenomenological-hermeneutic approach has informed not only the analysis but the entire research process, including what kind of knowledge is in our interest and what kind of questions should be posed to generate rich data that unfolds the lived experiences of people. In line with this perspective, we approached participants with an open and reflective attitude, inviting them to freely narrate and explore their lived experiences through both photographs and dialogical interviews. The emphasis was on participants’ own interpretations and meaning-making, rather than imposing predefined categories or frameworks [42]. We invited individuals in voluntary inpatient care in a new mental health facility in Norway and combined photovoice with individual interviews [38, 58]. We sought to gain a deeper understanding of how the patients’ sense of safety and well-being was affected by their care environment, including their personal space, and their lived experiences of inpatient care.

We adopted the photovoice methodology to generate data, as outlined by Wang and Burris [66]. Photovoice is both a method and a methodology originating in sociological research where three basic theories have laid the foundation for its development: liberating pedagogy inspired by Freire’s [15] ideas about knowledge and empowerment, feminist perspectives, and photo documentation. Photovoice can help reveal an enriched understanding of experiences by eliciting additional visual and narrative data in phenomenological inquiries [52]. Photovoice aims to give vulnerable and marginalized people a voice to articulate their needs and perspectives. It has been developed and used in many different research disciplines, such as psychology, nursing, and ethnography [50]. Photovoice was previously used to explore the meaning of the physical environment in forensic psychiatric settings [49, 51]. In the present study, a modified version of the photovoice methodology was used (i.e., the analytical process was performed by the researchers only). Inpatients were invited to take photos of their immediate surroundings, objects, and areas in the units that meant something to them and were connected to a sense of safety and well-being. The rationale for using a modified version of photovoice was to better accommodate the clinical setting, the vulnerability of the participants in a state of hospitalization, and the practical and ethical constraints of conducting research in a newly established mental health facility. While traditional photovoice involves participants taking photographs in their everyday environments over time, our adaptation focused on supporting participants to reflect on their lived experience within the institutional space, often within a limited timeframe and under guided conditions. This approach preserved the core participatory and reflective elements of photovoice, while ensuring emotional safety, confidentiality, and feasibility in a hospital setting. The modified method allowed participants to express perceptions of space, place, and care through both visual and narrative means, while being sensitive to the institutional context and the need for researcher facilitation. Drawing on principles from participatory action research, photovoice enables participants to critically engage with their own environments and bring to light the structural and institutional factors that affect their mental health and agency. In doing so, the method supports a form of bottom-up knowledge production where the patients contribute not only to personal storytelling, but also to a broader critique of systemic conditions.

### Setting—the case

This study is framed as a case study [68, 69]. NPK opened for patient intake during the spring of 2023 and has 80 inpatient rooms for adults and for adolescents aged 12–18 years. The units are closed wards and include the following: (1) the Psychiatric Emergency Unit, (2) the Subacute Unit/Psychiatric Intensive Unit, (3) the Unit for.

**Fig. 1:**
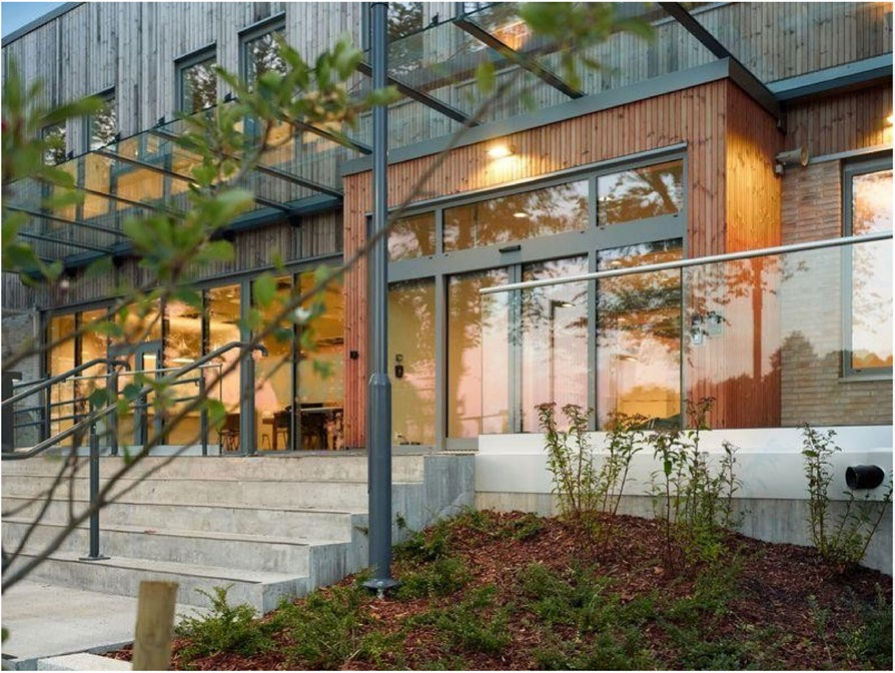
The main entrance by Marcel Tiedje

**Fig. 2:**
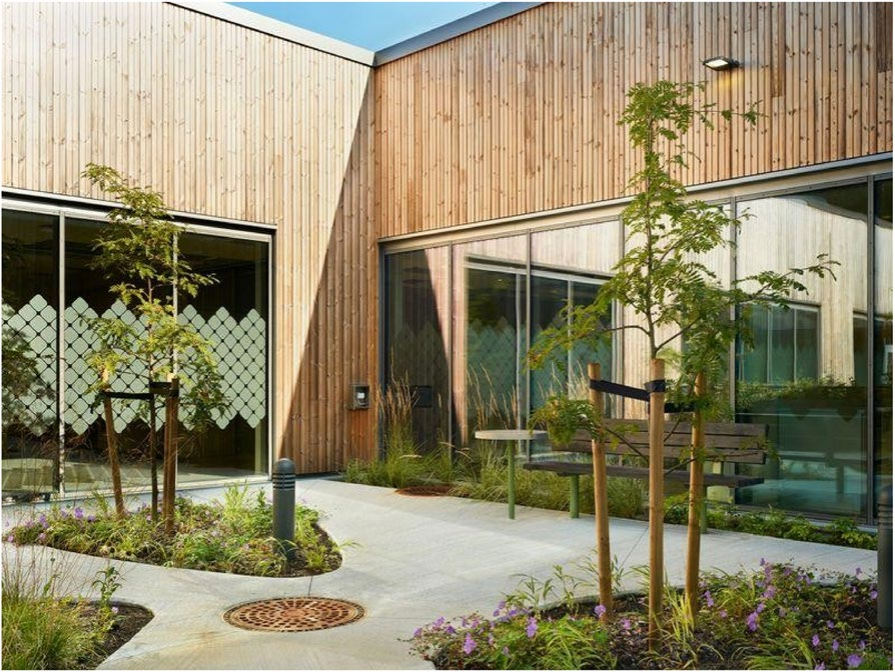
Atrium gardens by Marcel Tiedje

Assessment of Psychotic Disorders, (4) the Unit for Psychosis and Addiction Disorders, (5) the Reinforced Unit for Psychosis and Addiction Disorders, (6) the Forensic Unit, (7) the Unit for Geriatric Psychiatry and Cognitive Impairment, and (8) the Child and Adolescent Unit. The facility aims to use supportive design, natural elements, and access to nature to enrich care. All patient rooms are located on the ground level to ease the inside–outside flow. There are atrium gardens for all patients to freely access and silent zones with plants between and outside the patient rooms, reflecting a key feature is the strong focus on the therapeutic use of nature (Figs. 1 and 2).

### Participants

A purposive sample was applied, where patients admitted to the mental health facility [46] between August 2023 and April 2024 were invited to participate in the study. The participants were between 23 and 70 years of age with a mean age of 38. A few patients had previously received involuntary care, but at the time of the study, everyone was receiving care voluntarily. The sample comprised eight females and six males. Their demographic characteristics are presented in Table 1.

**Table 1:** An overview of the participant characteristics

Participant	Gender	Age	Educational level	Number of photographs	Unit	Interviews
1	M	44	Secondary school	3	1	In person
2	F	70	High school	4	7	In person
3	F	25	Secondary school	6	2	In person
4	M	23	Bachelor	3	3	Zoom
5	M	50	Secondary school	4	1	Zoom
6	F	35	Bachelor	5	1	Zoom
7	F	38	Bachelor	5	1	Zoom
8	F	59	Secondary school	3	1	Phone
9	F	32	Primary school	3	3	Phone
10	F	27	Secondary school	3	1	Phone
11	F	42	Bachelor	5	1	Phone
12	M	38	Master’s degree	4	2	Zoom
13	M	30	Primary school	4	3	Phone
14	M	24	Secondary school	0	3	Phone
Mean		38,4		3,7		

### Data collection

Data collection took place from August 2023 to April 2024. Several meetings were held to inform unit leaders and staff about the study. A contact person at each unit identified potential participants and extended the initial invitation by giving them the information sheet and time to consider the invitation. The participants were informed by the first or second author about the study and were provided with the opportunity to ask questions. Moreover, they were informed about their right to withdraw from the study without having to explain why. Written and oral consent was collected before data collection sessions commenced. None of the participants decided to withdraw from the study.

### Procedures

Data collection was performed in two steps: photo sessions and follow-up interviews. The contact person notified the researchers (AH and CRF) about the patients who had accepted the invitation. Thereafter, the researchers (AH or CRF) accompanied them while taking photos using a polaroid camera. The purpose of this was to ensure fellow patients’ integrity and to support the patients in using the polaroid camera. They were instructed to take photos of aspects of the surroundings that held meaning to them but avoid photographing other people. In our adapted version of photovoice, a researcher (either AH or CRF) was present and/or accompanied participants during parts of the process. This was done primarily to ensure emotional safety, ethical oversight, and to offer supportive containment in a context where participants as inpatients were navigating psychological vulnerability and in the institutional setting of a mental health facility. The presence of a researcher could help participants feel supported, validated, and encouraged — fostering a sense of being taken seriously. However, this presence may also have influenced the choices participants made, both in terms of what they chose to photograph and how they expressed themselves. It is possible that some participants exercised self-censorship due to perceived expectations or subtle dynamics of power, even when the process aimed to be participant-led. The presence of a researcher might have introduced a tension between authentic self-expression and the wish to please, or to avoid exposing aspects of their experience that felt too vulnerable or risky to share. At the same time, being accompanied may have enhanced participants’ sense of empowerment through relational support — especially for those who might have felt uncertain or overwhelmed by the task. The collaborative dimension, in this way, may have functioned less as surveillance and more as co-regulation, facilitating a process of reflection-in-relationship. Thus, while researcher presence inevitably introduces certain constraints, it may also have opened up possibilities for meaning-making, dialogue, and containment that might otherwise have been inaccessible — particularly in a therapeutic and institutional context. This duality — between support and influence, empowerment and possible constraint — reflects a central tension in participatory research i.e. the aim to foster voice and agency within a framework that remains inevitably shaped by institutional and relational dynamics.

After the photo sessions, the participants agreed to an individual follow-up interview with the first author (AH). Due to geographical distance, most of the follow-up interviews were conducted over the phone or on Zoom, except for initial three that were conducted in person. The participants were encouraged to talk about their experiences in relation to the research questions. Specifically, they were asked open-ended questions about the photographs they had taken that showed elements of the hospital environment that were important to them. In other words, they were asked to reflect, show, and talk about the physical environment. Their responses were followed up with questions like “Can you please tell me why you took these pictures and what they mean to you?” and “Please share how you have noticed that the environment affects you as a patient and the treatment experience.”

In line with photovoice methodology, participants had full autonomy in selecting what to photograph and which images they wished to discuss during the interviews. The researchers did not guide or influence the content of the photographs, and participants were free to withhold photographs they did not wish to share or talk about. This approach aimed to respect the participants’ agency and ensuring that the meaning-making process remained participant-led [66]. While participants selected the photographs for discussion, the final decision on which photographs to include in the paper was made by the research team, primarily to illustrate the themes that emerged during analysis. This selection process was conducted with care to preserve the integrity of participants’ perspectives while also meeting publication requirements.

A total of 52 photographs were taken by the 14 patients. The information and photographic sessions lasted approximately 20–30 min per participant. The length of the interviews ranged from 8 to 45 min, with an average duration of 24 min. The interviews were audio recorded and transcribed verbatim by AH. The authors selected the photographs included in the paper, based on their relevance to the analytical themes and their photographic quality. The photographic sessions were facilitated by the first and second author. Participants received oral and written information about the project, use of the Polaroid camera, ethical boundaries (e.g., not photographing people), and were invited to take pictures of meaningful aspects of their everyday life in the mental health facility.

### Rigor

To ensure the rigor of the study, the principles of credibility, confirmability, transferability, and dependability were established [40]. The research group worked closely to ensure that the analytical processes and the methodological steps were properly undertaken to ensure credibility. CRF conducted the majority of the photo sessions with patients, and AH conducted the interviews. The process of analysis was performed by AH and SO with continuous feedback from the research team. Reflexivity was maintained throughout the research process by actively reflecting on and discussing our preunderstandings as clinicians and researchers. Initially, we assumed that patients in a closed mental health facility would predominantly describe their environment as limiting or distressing. However, during the analysis, these assumptions were challenged by the participants’ descriptions of safety, beauty, and meaningful spaces. This reflexive process allowed us to remain open to unexpected meanings and interpretations in the material. The research team also reflected on the notion that a text always has a surplus of meanings, as suggested by Ricœur [54], emphasizing that a text always holds more than one plausible interpretation rather than one probable meaning [41, 42]. Furthermore, during interpretation, we strived to uphold internal consistency in relation to competing interpretations, as not all possible interpretations are equally probable.

Internal consistency was ensured by maintaining a transparent and iterative analytic process which was grounded in the hermeneutic circle. Throughout the interpretation, we moved continuously between the patients’ narratives, their photographs, and the developing themes to ensure that our interpretations were coherent and rooted in the data. Regular discussions within the research team allowed us to refine our understanding and check that the themes remained consistent with the lived experiences described by the patients.

### Ethical considerations

This study was performed in accordance with the Declaration of Helsinki [48] and ethical principles for medical research involving human subjects. An information letter was handed out to the participants, and they were given time to consider the invitation to the study. Informed consent was obtained before data collection process, and the voluntary nature of participation was emphasized. The participants were informed that they could withdraw at any time without any impact on their care.

To ensure the confidentiality of the participant data and their protection (privacy), the study was approved by the Norwegian Agency for Shared Services in Education and Research (SIKT). The project was submitted to the Regional Committees for Medical and Health Research Ethics (REK) in Norway, who in turn concluded that the project was outside the scope of health legislation. The project was also approved by the Swedish Ethical Review Authority, as the research group is partly based in Sweden.

## Analysis

The interviews were analyzed using a phenomenological hermeneutics approach inspired by Ricœur’s [54] philosophy and further developed by Lindseth and Norberg [41, 42]. Ricœur’s [54] philosophical theory of interpretation entails a dialectical movement between understanding and explaining a given text rather than treating these as two distinct approaches. Instead, understanding and interpretation are seen as complementary, representing a necessary and unified process to arriving at valid knowledge. In our analysis, the text comprised interview transcripts that served as “human testimonies fixed in writing” ([53], p. 160). The goal of interpretation is to uncover a text’s inherent meaning and to illuminate the direction it points toward—namely, its contribution to the ongoing discourse.

To arrive at an understanding, structural analyses are required to explain how an interpretation has been developed while ensuring the validity and objectivity of the scientific exercise. Accordingly, explanations focus on the formal, structural, and objective elements of a text. However, understanding represents “deep interpretation,” where an interpreter seeks to uncover the underlying meanings, intentions, and existential dimensions of a text. This entails the interplay between understanding *what* a text conveys, which requires an empathic approach and a sense of presence, followed by the adoption of an objective perspective to explain *why* a text communicates its meaning, thereby moving beyond an initial understanding. Ricœur [54] described this process as an interplay between proximity to and distance from a text. To achieve a valid interpretation grounded in a text and relevant to a study’s context (hermeneutics of suspicion), the text must be critically questioned and scrutinized [54].

The process of textual analysis involves, according to Lindseth and Norberg [41], a dialectical movement back and forth between the two stages of *naïve understanding* and *structural analyses* before arriving at a third stage: *comprehensive understanding*.

**Fig. 3:**
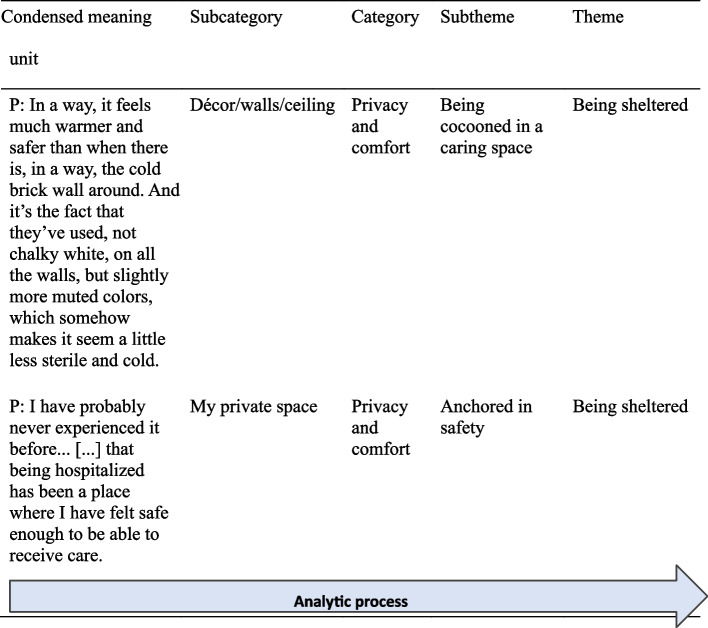
Overview of the analytic process from a condensed meaning unit to a theme based on the first and second rounds of structural analyses

A naïve understanding is a preliminary first interpretation resembling a qualified guess. It provides entry into the data and serves to direct the next step. Therefore, the interview transcripts were read and reread to grasp and understand what the text was “saying.” Thereafter, several rounds of structural analyses were performed to validate or invalidate our naïve understanding with a critical attitude. In seeking to (in)validate, both thematic and narrative approaches were used [41, 42]. A comprehensive understanding was finally reached by summarizing and reflecting upon findings from previous analyses pertinent to the research questions, in addition to the researchers’ preunderstandings/theoretical standpoints and relevant literature. This culminated in a deeper understanding that was novel. For an overview of the analytic process, see Fig. 3. 

## Findings

### Naïve understanding

In the following, our naïve understanding is presented, which constitutes the first step of the analytic process and is a qualified guess that is a way to enter the data [41, 42].

The new mental health facility is understood as a place where patients feel genuinely cared for and as a space someone had thoughtfully designed with patients’ well-being in mind to support patients in a time of personal crisis and increased vulnerability. The facility is felt as a place where *care* is manifested and where design made a significant difference; however, it is difficult to make these feelings explicit. Being “there” fosters a sense of dignity and engagement, making positive distractions more accessible. In addition, the facility contributes to feelings of being *worthy* and being able *to rest and feel safe.*

Encountering the new facility is a transformative experience—it feels less like a traditional hospital, shedding the “sterile and cold” atmosphere in favor of one that is “warmer and safer.” Dwelling there at a time of vulnerability means that it is easier to find a sense of calmness, inner peace, and hope. The environment thus materializes as a place that holds a promise of being unburdened and supports patients’ *being in there.* Subsequently, the environment supports patients’ ability to engage with the care provided. Simply gazing into nature through a window offers psychological respite—a moment of calm to reflect, process emotions, and gain a sense of stability amid times of crises.

### First structural analysis—describing the facility’s spatial environment

This step in the analytic process is characterized by an objective/structural or critical attitude to explain our naïve understanding [41, 42] to describe “the what-ness” of the text [54]. During this first round of structural analysis, the interview transcripts were read repeatedly. Meaning units were identified and expressed as briefly as possible in everyday language and then condensed. Thereafter, the condensed meaning units were sorted according to similarities and differences to organize the data before developing subcategories and categories (see Table 2). We also organized the photos to align with what was narrated in the text.

**Table 2:** Overview and categorization of patients’ photos—first structural analysis

Number of Photos	Subcategory	Category
8	My private space	Privacy and comfort
6	Windows and view outside	
9	Décor/walls/ceiling	
6	Surrounding nature/atrium gardens	Shared spaces
3	Gym and sports area	
2	Café/vestibule	
18	Living room, kitchen, and other common spaces	

The photos were then examined to grasp their possible meanings in relation to the participants’ narration/stories and sorted into different categories as a part of the analysis (Table 2). The authors strove to control their respective preunderstandings so the text could be considered as objectively as possible.

### Privacy and comfort

The patients’ private rooms are painted in soft and soothing colors; they feature wooden materials and a ceiling with sound insulation (sound proofing). The blinds offer privacy when wanted and a view outside when that is preferred. Patients can sit near the window and gaze out onto nature through large windows in their rooms; the participants appreciated having something “nice” to rest their eyes on. The participants also valued artwork on the walls, as they served as positive distractions. Personal belongings and photos served as reminders of ordinary life outside of the hospital. The patients also valued having a bench for visitors and staff to sit on, as this meant they did not have to sit on patients’ beds, which feel more like private spaces. Having a comfortable bed that was more “ordinary” in appearance than a hospital bed made relaxing easier. Relaxing in one’s own room was also perceived as easier than in the common areas, and patients found it a positive experience to have the opportunity to retire to their rooms when needed. Furthermore, having the opportunity to do enjoyable activities in their private room was much appreciated.

### Shared spaces

**Fig. 4:**
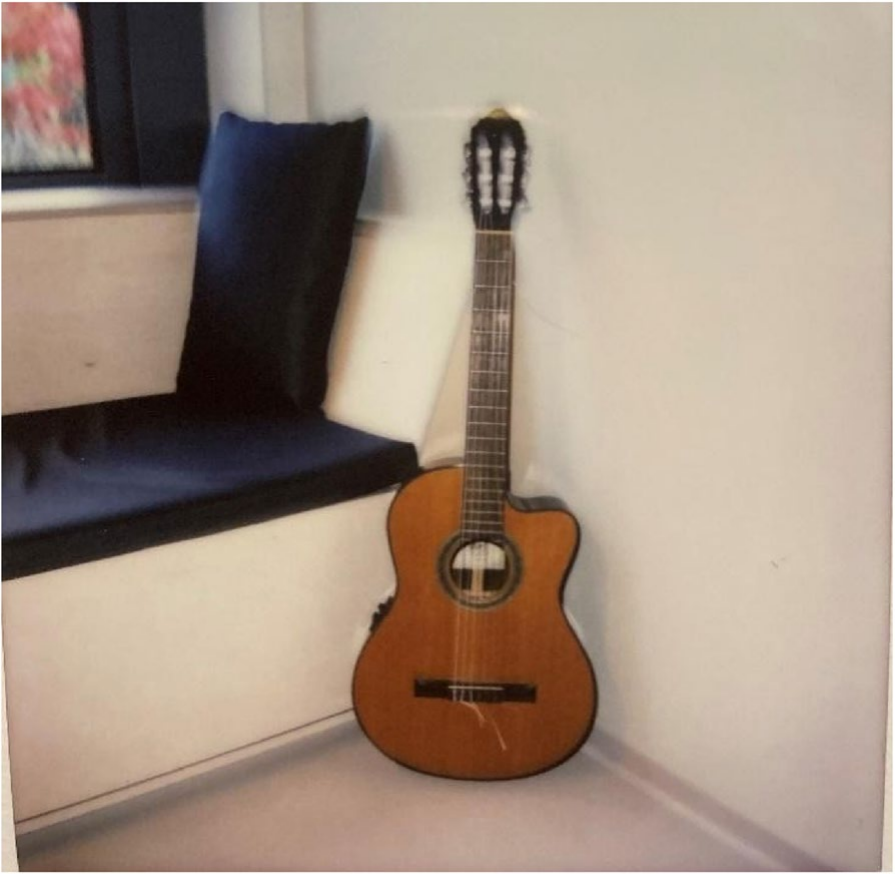
My private space – Privacy and comfort. Photo taken by a young male showing the bench in his private patient room—a place where he enjoys playing the guitar

**Fig. 5:**
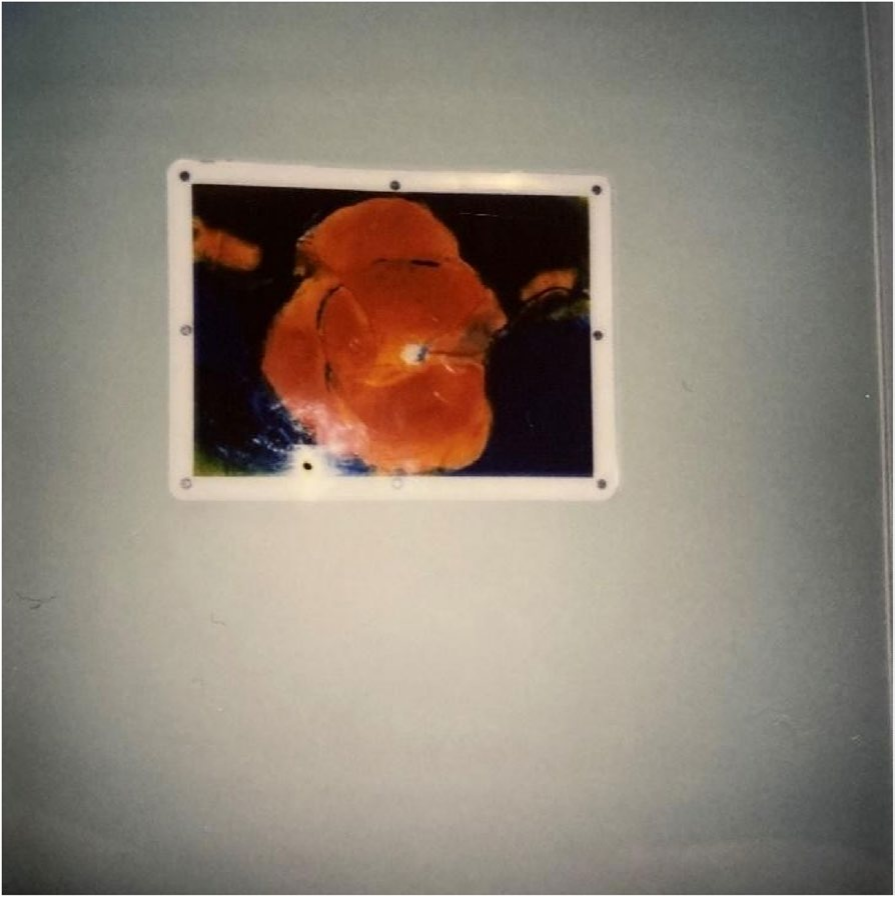
Décor/walls/ceiling – Privacy and comfort. Photo taken by a male showing the artwork/photograph on the wall in his room that he looked at to experience positive distractions

**Fig. 6:**
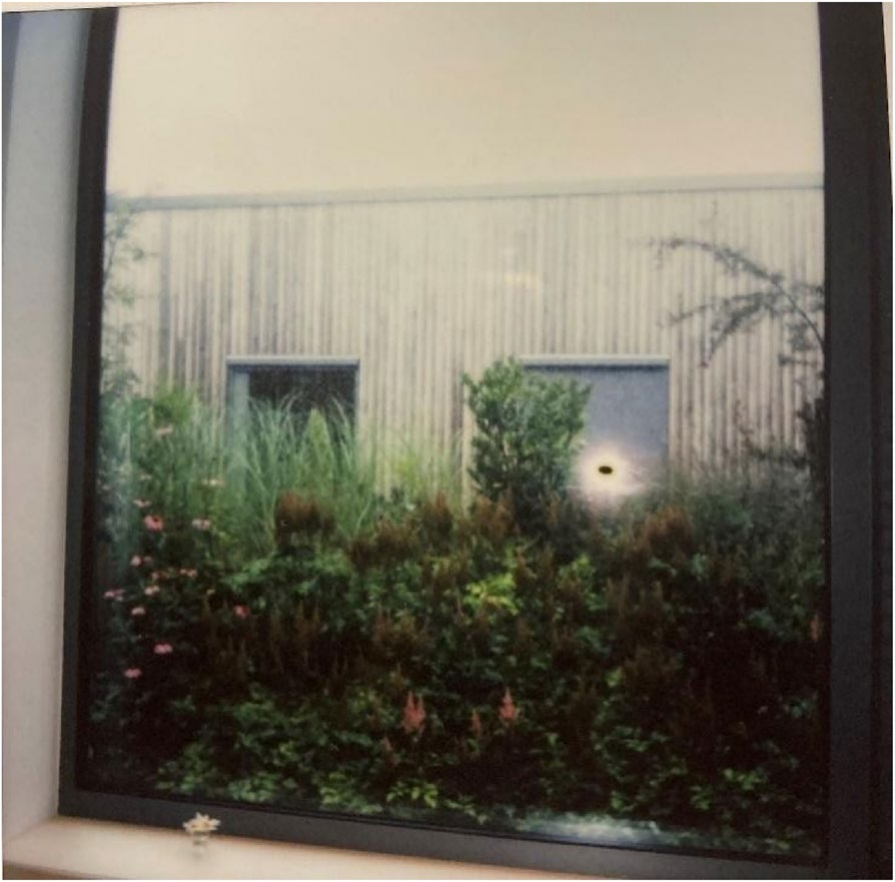
Windows and view outside – Privacy and comfort. Photo taken by a young female showing the view of nature outside that she enjoys from her room

**Fig. 7:**
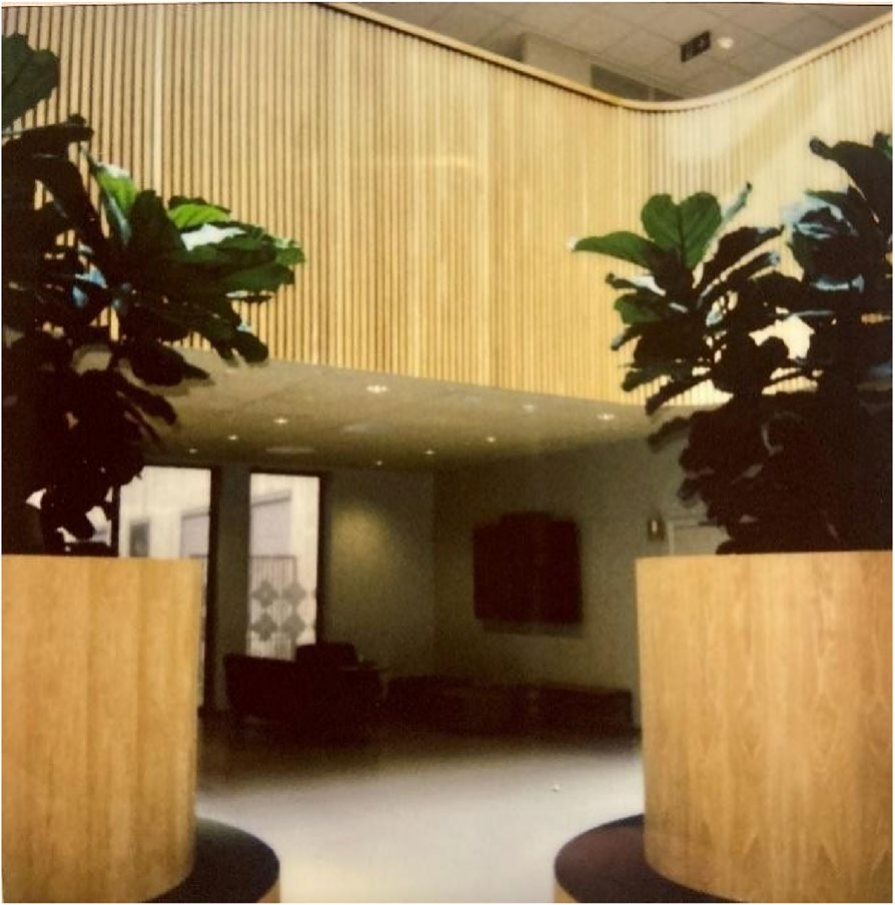
Café/vestibule – Shared spaces. Photo taken by a male depicting the café/vestibule with a “welcoming entrance” in the common public area

**Fig. 8:**
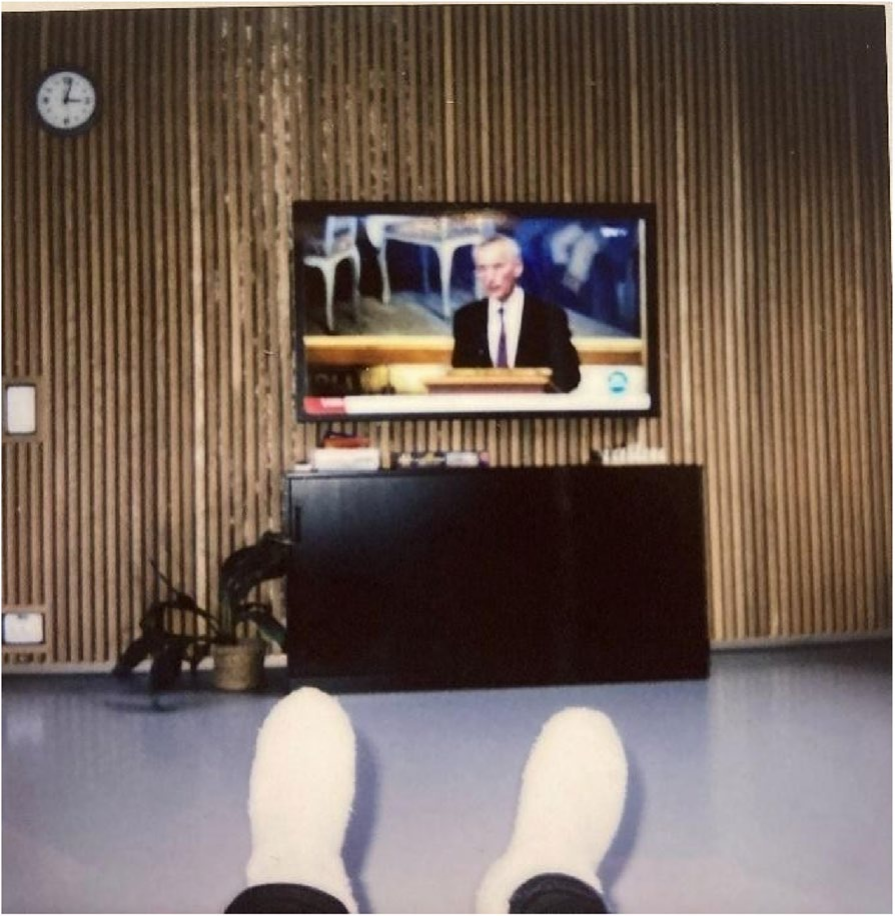
Living room, kitchen, and other common spaces – Shared spaces. Photo taken by a young male showing he is watching television in the living room in the common area for patients

**Fig. 9:**
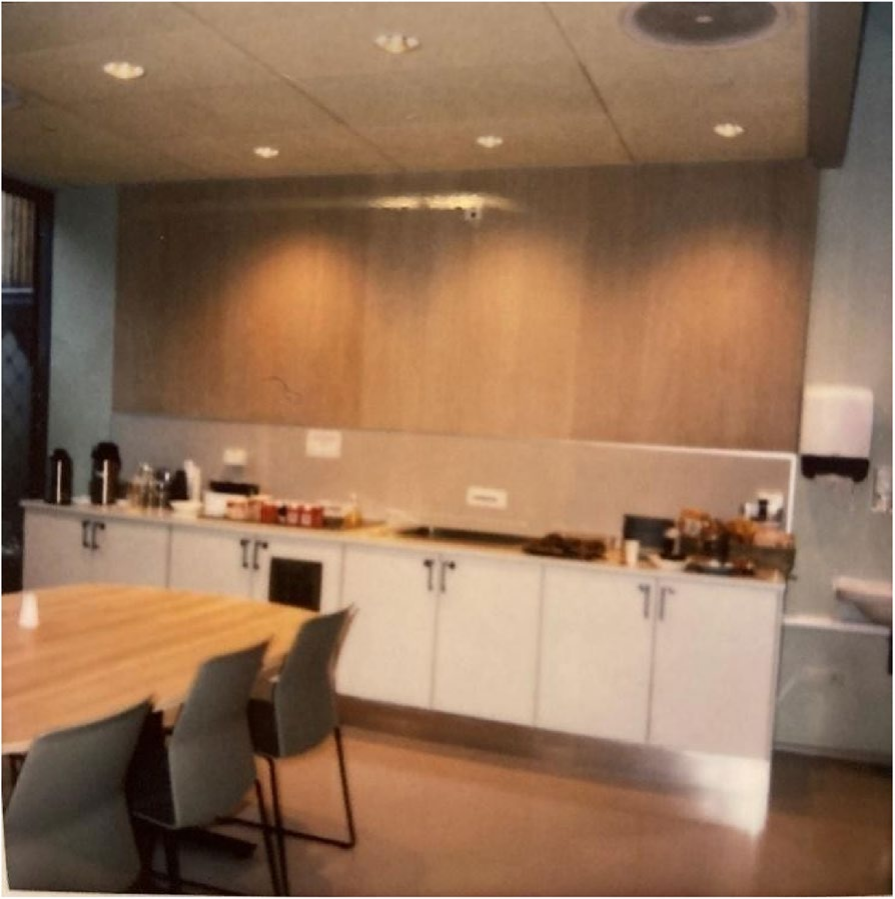
Living room, kitchen, and other common spaces – Shared spaces. Photo taken by a young female showing the kitchen in the heart of the unit, which is considered a gathering point

“Open spaces” in the common areas enable interactions and foster a sense of community with others, including patients and staff. Having a living room with television provides the opportunity for company and a distraction from issues. The living room and kitchen feature opportunities for shared activities. For example, patients can play a game, bake, or cook. The kitchen table reminded participants of tasty food and companionship and acts as a gathering point. Atrium gardens and nearby nature were greatly appreciated by the participants. However, some patients did not feel safe when they had to share atrium gardens with another unit. Moreover, patients desired “fresh air”; sometimes, there was a lot of cigarette smoke in the atriums, which was annoying and deprived them of the opportunity to get fresh air. The training room and gym were frequently used and are considered tools in recovering from mental illness. The public café/vestibule with its friendly and welcoming atmosphere was highly valued, as it offered some “normalcy” and refuge from the unit and sometimes from being in a state of crisis. As in a somatic general hospital, the café/vestibule in this new mental health facility was accessible to everybody. This also increased normalization by offering a glimpse of “ordinary life” outside and reduced stigmatization (Figs. 4, 5, 6, 7, 8, and 9).

### Second structural analysis

The findings of the first round of structural analysis revealed a need for a deeper understanding of the meanings of the mental health facility. By following Ricœur's [54] theory of interpretation, a next step is to explain and unfold meanings connected to the phenomena under investigation. In this step, the text is again scrutinized, and its “how-ness” in relation to what the participants spoke about is examined. Therefore, we performed a second round of thematic structural analysis focusing on meanings (i.e., how the care environment contributed to the participants’ safety and well-being). Three themes emerged from the interviews. Table 3 provides an overview of themes and subthemes in the second round of structural analysis.

**Table 3:** Overview of subthemes and themes – second structural analysis

Subtheme	Theme
Anchored in safety	Being sheltered
Being cocooned in a caring space	
Being lost in space and time	
Affirmation of worthiness	Feeling cared for and dignified
Being genuinely cared for	
Finding a way forward	
Inner harmony in the therapeutic landscape of nature	Being held by nature
Striving for normalization	

### Being sheltered

Being sheltered captures a profound human experience of finding solace and security within one’s surroundings. It embodies a state of anchoring amid vulnerability, offering a sense of containment and care. Yet, this experience is multifaceted—ranging from feelings of being cocooned in a nurturing space to moments of disorientation where time and space dissolve.

#### Anchored in safety

A sense of safety is not merely the absence of physical and emotional threats but also the presence of nurturing, understanding, and supporting elements. As the patients dwelled in the mental health facility and spent large amounts of time there, they perceived it as essential to feel emotionally safe and physically secure there: *“I have probably never experienced it before […] that being hospitalized has been a place where I have felt safe enough to be able to receive care”* (P3). This was especially true when care is involuntary. It became even more important that the environment resolved some tensions between resistance and acceptance in the event that a patient isn’t participating in care voluntarily. Furthermore, having objects in the environment that fostered a sense of familiarity made them feel safer. Objects have the power to connect a current situation to memories and places and create psychological and physical safety, as well as a temporary escape and suppress the feeling of alienation in a “new” and strange place. A patient explained that in his patient room, there was a picture of a landscape similar to the one he had grown up in. The picture evoked positive feelings of the landscape that he knew. His safe space at home was not physically present, but a picture of something that resembles something “of the same” helped him remain calm and dare to be present in the current situation: *“And in the state I was in, there was a sense of security in that”* (P5).

#### Being cocooned in a caring space

Being hospitalized in a mental health facility could be a scary experience. In contrast to previous experiences in mental health facilities that evoked feelings of confinement, claustrophobia, and sickness, exacerbating patients’ vulnerability, the new environment created feelings of being enveloped in safety. The sound proofing and the quiet common areas increased the patients’ sense of safety, as they felt negatively impacted by noise and outbursts: *“You feel a little more like wrapped in cotton wool”* (P3). Being enveloped in safety gave patients a feeling that external pressure had been softened, and the patients experienced being cradled by an environment that offered both physical and emotional protection. As these spaces fostered a sense of safety and security, patients’ ability to engage in therapeutic conversations improved. They emphasized the contributions of the environment that fostered a sense of trust, allowing for rest and reprieve from vulnerability: *“The colors make it safe and warm. The décor makes it safe and warm. That’s nice. […] I find it easier to talk about things when it is safe and warm around me”* (P6).

Some of the patients were confused and anxious, and some where psychotic at the beginning of their admittance. One patient put it this way: *“Although it was a kind of prison, I felt safe at the same time. That’s what I needed to feel. I needed to feel a physical structure around me and feel safe”* (P7). The therapeutic settings and the feeling of being cocooned facilitated healing and recovery by creating a foundation of stability, where the patients felt held and supported as they navigated their personal journeys.

#### Being lost in space and time

Struggling with mental health issues and the acuity of mental illness may also mean experiencing a state of disorientation and disconnection from familiar anchors, where the boundaries of physical and temporal realities blur. The data showed that the environment needs to “carry” and “hold” patients during times of confusion or inability to feel or regain control. This means being dependent on the staff and not having the ability to orient oneself toward normality and routines. Here, the environment had an effect on how the patients could navigate and create control. For example, not having a clock and a calendar (date) in the patient room made the same patient feel confused when she was previously in a psychotic state: *“And then I was very confused that there was no clock there. I don’t know why there is no clock or date in the room. It really stressed me out not knowing what time it was and what date it was”* (P7).

### Feeling cared for and dignified

Care, concern, and dignity can neutralize the shame that may arise from having mental health issues. This reflects a deep human need for support and belonging, encompassing moments where worthiness is recognized, care is extended, and a sense of calm emerges from being embraced in a supportive environment, fulfilling the yearning for connection and respect. In essence, people require existential consolation.

#### Affirmation of worthiness

At this new facility, the feeling of being less of a patient and more of a person is central. The facility emphasizes being an individual and not a diagnosis. Patients often experience a stream of negative thoughts toward themselves. Relating to these thoughts is difficult and makes emotional regulating challenging. Some patients had been admitted numerous times to other facilities that were “outdated”. As such, some of them had a strong feeling that no one could help them, least not themselves. Sometimes, patients’ self-esteem was so low that they had previously felt hopeless in therapy because they felt they were not worth it. In the new environment, which felt so incredibly soothing to them, a lot changed, most importantly their self-worth and genuine will to receive therapy: “*It made me feel, that I can feel that even though I am sick, I am strong in some ways, as it were. That I can have a better self-image, or self-respect. That you manage to achieve something”* (P11).

#### Being genuinely cared for

Patients found the new mental health facility to have a significant calming effect. This was directly connected to the environment’s qualities, such as sound isolation and the building’s design. Being in a calmer environment also made reflection easier, and ability to receive therapy was therefore improved: *“I felt incredibly cared for, and I wish, I wish I had sought help sooner (cries) and not been so scared”* (P7). For these patients, the new environment promoted stress reduction and mental restoration. Some of the patients thought that this increased the speed of recovery: *“I had a little recovery process in there [patient room], which helped me because… It was the way it was; it wasn't such an old psychiatric institution”* (P5). Another patient reflected on what influenced her the most: *“So, what’s around [the environment] is pleasurable when you’re in a bad phase [psychological state] yourself. I think that is what has meant the most to me […] faster recovery I think”* (P6).

The physical environment communicated that “someone” had thought about what a “good” healing environment should look like, demonstrating an understanding of what makes a building conducive to patient well-being, comfort, and care. Another patient shared, *“But here, it was like, in this building, I felt like it did everything, and I felt a lot healthier, and I felt like a human being. I started to have hope again, to be honest”* (P7).

#### Finding a way forward

The built environment enabled possibilities to regain resources because it promoted feelings of calmness and belonging, helping patients face their challenging situations. A patient was reflecting and trying to put into words how the new facility had enabled “new” thoughts to emerge. These thoughts meant new opportunities that they had never thought of previously. This meant entering a new landscape of possibilities and regaining resources:

*“The main essence of it is really just that for me; it was easier to lower my shoulders and find a calm, which also meant that… it was easier to think in a different way when the environment around me facilitated it”* (P6).

There was a distinct feeling of being in a hotel room. The environment presented itself as a symbol for safety and a pause from patients’ current situations, creating comfort and empowerment: *“So it felt like I was in a hotel; I felt privileged”* (P7). The patient room became the patient’s own room for a while because they dwelled in the room. They valued the room for its atmosphere and the respect it signaled, and it helped the patient find a way forward.

### Being held by nature

Evoking a profound sense of connection and restoration, the natural world offers a therapeutic presence. Fostering harmony and inviting individuals into a healing landscape can sooth and nurture by delving into interactions with nature. This thoughtfully designed setting contributed to emotional balance and well-being by illuminating the interplay between nature and design.

#### Inner harmony in the therapeutic landscape

A sense of emotional balance, mental clarity, and calm that aligns with experiencing peace of mind was experienced as an internal state of being that freed the participants from distress or worry, even if just for a moment. The atrium gardens and nature revitalized them: *“Just being out in nature gives me a lot. And I somehow get more energy from it. And I feel that I somehow achieve something when I go for a walk”* (P4). Additionally, the view of nature was much appreciated to gain calmness: *“No, it’s just that I’ve been sitting a lot looking out of it [the patient room window], so I wanted to take a picture of it. It’s wonderful.*

*You can look out toward the forest, for example. It gives me a lot. It gives me peace”* (P13).

There was a contrast between being *inside* and *outside*. In the atrium garden, patients gazed at nature, green plants, flowers, insects, and feeding birds. This was a positive contrast to inside, where a lot of things happened constantly. Outside, there was freedom and pause: *“It was just like, when you’re isolated, it’s just like a bird means everything to you. Do you understand what I mean? […] Animals can heal quite a lot”* (P7).

Being in nature curbed patients’ stress and made it easier to open up. As such, being in nature enhanced their readiness to pursue the treatment process: *“I have had a lot of selftherapy there. In nature. It’s just being in it and noticing the little things in nature. Mm… therapeutic”* (P12).

#### Striving for normalization

Patients’ “being in there” restored feelings of normalcy and stability. Within a mental health facility, the therapeutic process is often gradual and supported by spaces and interactions that promote safety, autonomy, and connection. By fostering a physical environment that feels both ordinary and supportive, patients are empowered to rebuild their lives, reconnect with “themselves” (their identity), and hopefully find a renewed sense of purpose: *“First of all, the bench that is there, because I like to stay in the [patient] room for a while and being able to be there without sitting in bed, it’s a bit like a normal place to sit and gaze out the window”* (P3).

Regarding environments designed to support therapeutic work, a patient claimed the following: *“I think for my part, it was like when I woke up, I thought that at least it’s nice [the physical environment] when you’re having a hard time. And not just focusing on the fact that I feel bad, or that the situation that has happened is bad. Then there is the fact that there was an automatic connection that today is a new day. It’s nice to open your eyes even if it’s tough”* (P6). She experienced that when she or the staff opened the blinds, being able to look out at nature evoked feelings of normalization. This reminded her that there is life “outside” the facility and that “life goes on”; it has a rhythm, and she is part of it. This reflects a deep desire to reclaim a sense of equilibrium and familiarity amid mental chaos and distress.

### Comprehensive understanding

The final step in the analysis process involved the formulation of a comprehensive understanding [41, 42]. This step finalized the process of arriving at a valid interpretation based on the naïve understanding, the structural analyses, the context of the study, and our research questions. According to Ricœur [54], a comprehensive understanding is a contribution to the ongoing discourse, in this case that on mental health and patients’ lived experiences of being in there. An interpretation mediates a message, creating room for understanding and intelligibility with a forwarding orientation. This fusion of horizons occurs between an interpreter’s perspective and the text’s perspective. Our comprehensive understanding of the patients’ lived experiences in this new mental health facility can be interpreted as a journey of finding a sanctuary within the interplay of care, nature, and the patients’ selves—the self. It is a profound connection between the physical environment and patients’ emotional and existential well-being, meaning that care cannot be separated from where it is exercised. Drawing on Kari Martinsen’s [45] *Care and Vulnerability*, the patient room can be seen as a liminal space that symbolizes the transition between illness and regaining health, autonomy and dependence, and hopelessness and hope. Martinsen [45] claimed that a caring environment may sing different songs of happiness, of sorrows and pain, and of struggle while being vulnerable. The environment should “hold the patients,” as in this space, patients often confront existential questions, making a room’s atmosphere particularly significant. By designing environments that “sing” songs of compassion, dignity, and connection, then and only then will society have achieved humanity at the heart of care. Heidegger [24] claimed that a place to dwell should connect humans to “sky, divines, earth and nature”—a fourfold of elements. Nature, access to the outdoors, and a facility’s design should connect patients to the world outside and bring forth the fundamental elements needed to find a way forward and strengthen peoples’ well-being. Given this comprehensive understanding, there are new opportunities to realize their life projects. Moreover, several participants described feeling calmer and more “mentally free” when seated by a window or when exposed to natural light. These descriptions resonate with Ulrich’s [61] theory of supportive design, which emphasizes the role of nature views and daylight in reducing stress and promoting recovery in healthcare settings. When participants described moments of relief or containment in specific areas of the facility — such as near windows or spaces with natural elements — their experiences can be interpreted in light of Ulrich’s theory of healing environments, where the physical surroundings support emotional regulation, orientation, and a sense of safety. Their meaning-making aligns with what Ulrich identifies as restorative environmental features, which may act as non-verbal therapeutic agents.

## Discussion and reflection

In this study, we sought to understand and interpret the meanings of the care environment and its contribution to inpatients’ sense of safety and well-being. We focused on the lifeworld as a context for understanding what it means to be a patient. Research interviews were combined with images, which generated detailed data and a deeper understanding of patients’ experiences. Photovoice enabled us to enter the lived experiences of the patients from their own perspectives. The new mental health facility appear to provide a protective buffer, enabling patients to disconnect from external, and sometimes internal, pressure while navigating difficult and demanding feelings. These moments of grounding created a foundation for recovery and enabled the ability to receive therapy. The extent of the positive findings was surprising for us. Prior to data collection, we anticipated that participants could focus on negative aspects of the institutional environment, reflecting experiences of restriction, disconnection, or discomfort — themes commonly found in both clinical practice and existing research on mental health facilities. While such elements were present, we were struck by the extent to which participants mainly described positive experiences, such as moments of calm, safety, and personal meaning embedded in everyday routines or physical surroundings. This challenged our initial assumptions and highlighted the importance of approaching the data with openness and sensitivity to the unexpected, as emphasized in phenomenological-hermeneutic methodology. It also underscored the need to hold space for complexity in participants’ lived experiences — where difficulty and healing often coexist.

The biophilic design of the physical environment, coupled with supportive care, succeeded in facilitating moments where the patients felt truly seen, valued, and connected. However, communal life in the shared space was experienced as a dynamic interplay of tension between patients’ differing needs and wishes. As a result, various dilemmas arose.

Integrating nature into the facility made a difference for the majority. The facility’s outdoor areas offered a green oasis that sometimes tempted them to go outside and take a pause—maybe disappear into a respite. Nature also enabled the discovery of new thoughts, sometimes leading to new paths forward in life. The latter should also be understood against the cultural traditions of the Nordic countries. Love for nature is embedded in the Norwegian people’s heritage and culture.

In a related study, Hagerup et al. [20], we examined the expectations of the project group involved in planning and designing the new mental health facility. Their intentions were to create a supportive and natural design that allowed for autonomy, integrity, and normalization, as well as flexibility to address individual needs. Furthermore, the design prioritizes individuals and their dignity. The results of the present study revealed that the project group was successful in achieving a supportive and natural environment where patients felt a sense of safety and well-being. However, in another related study [21] that explored the new facility from the perspective of the psychiatric staff, identified a tension between patients and staff who constantly face conflicting priorities.

Well-being and a sense of safety are fundamental to care in the field of psychiatric care [23]. A sense of safety refers to a psychological feeling of being safe—a state of mind that is distinct from physical security—yet it is not limited to psychological and emotional safety alone [13]. In the context of patient care, psychological safety may play a critical role in promoting health and well-being and facilitating recovery [65]. Psychological safety is therefore essential within mental health care for inpatients, as it enables both patients and their families to convey suggestions and concerns [26].

The onset of a mental disorder can act as a sudden halt – interrupting the person’s life narrative and challenging their previous sense of self and direction. The mind has become unfaithful and a fierce enemy, leaving a person powerless. Ordinary life is paused and can be experienced as a broken promise of future health. Another life may take over, leaving a person with an unfamiliar identity. Having a mental disorder can be experienced as a person’s self-image being changed, which is often accompanied by intense emotions and sometimes the perception of a distorted world. Lack of self‐control can feel like a tsunami, and life becomes a shrinking box; feelings of being misunderstood and rejection arise [16]. Mental disorders can alter the affected person’s perception, implying that the environment needs to accommodate this. Being ill also changes and heightens the senses, leaving the person more vulnerable [6]. The voices of patients with mental disorders have occasionally been silenced [57]. Therefore, expanding our insights into their lifeworld is crucial for improving treatment conditions.

When the outside world is felt as an unsafe place, only offering chaos and unpredictability, feelings of rest and peace of mind become difficult and sometimes impossible. However, taking into consideration the voices of patients and helping them access spaces that feel safe and supportive could transform their inner world and help them achieve calm and tranquility, at least temporarily. The restorative effect of supportive and biophilic design, as well as outdoor environments, is well known [5, 29, 30, 64]. Access to nature and the outdoors and supporting design seemed to amaze patients. As such, environments that emphasize biophilic design could hold promise of future health. It is not a coincidence that supportive spaces that evoke familiar and safe memories and feelings can tap into images of being protected. A trusted environment immersed in “safeness” represents a welcome urge to recover and return to life outside. Such design has the potential to at least temporarily erase the remainders of their troubled minds and be a bearer of hope. The participants experienced glimpses of hope in the new facility. This was especially the case for those who had been admitted previously to older facilities. At the new facility, they experienced well-being, and future possibilities became more accessible [18, 39]. This implies a wanted or valued way forward that may be able to identify a hope for returning to ordinary life outside the facility. The person might be energized and motivated in ways that entail a sense of purpose.

As some patients had previously been admitted to older and outdated facilities, they were able to compare their experiences in the older facilities with those in the new facility. They experienced the new facility’s environment as a totally different one, finding it to be positive and constructive. “Hiding” the old and “outdated” buildings and the people in them may lead to increased feelings of shame and indignity. Dignified care therefore calls for more humanized health care where patients’ needs are addressed in a tailored manner and where the environment takes patient needs into account. Moving toward dignified care is a symbol that patients and mental health are worth focusing on. Dignified care is connected to feeling safe and secure and being treated as an individual and not “just a patient” [37].

Physical environments have been proven to reduce aggression and seclusion [58], which are often connected to stress. Supportive and biophilic design contributed to reducing stress and restlessness, which in turn strengthened patients’ ability to participate actively in treatment. At the same time, these outcomes were informed by individual differences and previous experiences, which may have influenced how the patients were experiencing their surroundings.

From a philosophical perspective, it is important to acknowledge that meaning structures or horizons contribute to the constitution of a phenomenon, which in this case was lived experiences of the built environment. It can be difficult to isolate the influence of the physical environment from other elements that affect patient outcomes, as patients are dependent on the quality of care, therapeutic relationships, and the culture of an institution. These elements have an influence on their attitudes toward being in a facility and experiencing it. Similarly, Modini et al. [47] emphasized that the physical environment is not the only factor that might affect patients’ experiences of the physical environment.

In the spirit of ecological caring [11], patients’ sense of safety and well-being emerges not only through professional encounters but also through the wider life-world in which care is embedded. From the patient perspective, the environment is experienced as part of care itself—shaping how one feels held, oriented, and acknowledged as a whole human being. This aligns with anthroposophic approaches to health and care, which emphasize the interrelation of body, soul, and spirit, and the importance of nurturing surroundings that respect human dignity. A mental health facility that integrates supportive and natural elements may therefore be understood as more than a neutral backdrop: it becomes a living context of care, co-creating conditions for trust, calm, and the possibility of inner restoration.

How patients experience safety and well-being is highly subjective and influenced by their personal backgrounds, mental health conditions, and cultural factors. This raises the dilemma of how to generalize findings to inform broader design solutions, as there does not exist one building that fits every need. How can future research balance individual, context specific insights that can guide future design and care practices across diverse settings?

### Methodological strengths and critique of method

In this study, we employed a phenomenological hermeneutic approach based on Ricœur’s [54] philosophy to understand and interpret text. Drawing on his philosophy, a given text always has a surplus of meaning, meaning a multitude of interpretations is possible. Therefore, our interpretation is only one plausible reading. Ricœur [53] claimed that *“it is always possible to argue against or for an interpretation, to confront the interpretations, to arbitrate between them, and to speak for an agreement, even if an agreement remains beyond our reach”* (p. 213). However, the structural analyses laid a foundation for arriving at what we consider a valid interpretation. We approached the text in two different ways, understanding and explaining, while striving to remain aware of our preconceptions and previous understandings. As researchers, we approached the material with prior clinical and theoretical understandings related to mental health, therapeutic relationships, and institutional care. Our backgrounds include experience in psychiatric practice, environmental psychology, and health care sciences, which inevitably shaped our initial expectations. However, in line with a phenomenological-hermeneutic approach, we sought to remain reflexively aware of these preconceptions throughout the research process. Engaging with participants’ photographs and narratives challenged us to attend more closely to the subtle, embodied, and often overlooked aspects of the environment that carried significance for patients — such as moments of quiet, access to natural light, or the personal meaning embedded in small routines. This process gradually shifted our understanding from a primarily structural and clinical perspective to a perspective that was more grounded in lived, sensory, and emotional experience.

Using photovoice gave patients a voice in the research process that could empower them to express their own perspectives in a creative and authentic manner. Photovoice can therefore be a method to promote a higher degree of authenticity by creating opportunities for participants to provide meaningful data. This is because the participants could control the nature of the data [52]. Interviews as a method have been criticized for not always accurately reflecting what is most meaningful to those experiencing a phenomenon [7]. As such, photovoice can make important contributions by providing health researchers and health professionals with the possibility of accessing the world from the viewpoint of the people who lead lives that are different from those traditionally in control of means [15, 56]. We used photovoice to elicit data that might deepen the understanding of patients’ lifeworlds and lived experiences of their care environment in an actual care setting. In using photovoice, our aim was to unveil and deepen our understanding of patients’ experiences of the environment and its relevance for their sense of safety and well-being. Phenomenological hermeneutics sought to understand their lived experiences, while photovoice not only captures individual experiences and perceptions but also makes visible the social structures that shape those experiences. By inviting participants to photographically document and reflect on their environments, routines, and encounters, the method can reveal how power dynamics, institutional norms, spatial arrangements, and social hierarchies are embedded in everyday life. In this way, photovoice acts as a participatory tool that helps illuminate both personal meanings and the structural conditions that may support or constrain well-being. Moreover, photovoice often seeks emancipatory change, whilst phenomenology makes no such claim [52]. Therefore, phenomenological hermeneutics and photovoice may be seen as complementary approaches.

Considering that photovoice as a method uses participant photography for knowledge development, this approach is also associated with a number of ethical concerns, such as privacy, safety, advocacy, ownership, and copyrights [14, 19]. To limit potential harmful consequences in a hospital environment the participants were escorted by one of the researchers when they engaged in photography to decrease the possibility of them taking photos of other patients. Disadvantages of using photovoice may include issues pertaining to personal judgment, such as who took photographs, what a user photographed, what a user chose not to photograph, and who selected which photographs to be discussed [66].

## Conclusion

This study uncovered the profound impact of thoughtfully designed mental health facilities on patients’ experiences of healing and recovery. The results revealed that the physical environment plays a crucial role in fostering safety, connection, and restoration. Furthermore, the results demonstrate that patients perceive design not merely as a functional element but as deeply supportive of their emotional and existential needs. The integration of nature, calming spaces, and supportive care creates a unique therapeutic environment that promotes grounding, self-discovery, and normalization. The insights from this study underscore the importance of prioritizing design features that address the various and holistic needs of patients within mental health care. Purposefully built mental health facilities that combine physical protection with emotional nourishment have the potential to become sanctuaries where patients feel not only safe but also valued and empowered to heal. The experiences of the patients in this study contribute to the growing understanding of how supportive environments influence mental health care and invite further exploration of the ways supportive design, nature, and humans’ interactions intersect to transform the patient experience.

## Data Availability

Our data cannot be shared openly, to protect study participant privacy. The participants in this study are patients voluntarily admitted to a mental health facility and they were promised confidentiality (anonymity) before participanting in the present study.
